# Myth or Reality—Transdermal Magnesium?

**DOI:** 10.3390/nu9080813

**Published:** 2017-07-28

**Authors:** Uwe Gröber, Tanja Werner, Jürgen Vormann, Klaus Kisters

**Affiliations:** 1 Akademie für Mikronährstoffmedizin, Zweigertstr. 55, 45130 Essen, Germany; kisters@annahospital.de; 2 IPEV Institute for Prevention and Nutrition, Adalperostr. 37, D-85737 Ismaning, Germany; werner.tanja@protina.de (T.W.); vormann@ipev.de (J.V.); 3 Medizinische Klinik I, St. Anna-Hospital, 44649 Herne, Germany

**Keywords:** magnesium, transdermal

## Abstract

In the following review, we evaluated the current literature and evidence-based data on transdermal magnesium application and show that the propagation of transdermal magnesium is scientifically unsupported. The importance of magnesium and the positive effects of magnesium supplementation are extensively documented in magnesium deficiency, e.g., cardiovascular disease and diabetes mellitus. The effectiveness of oral magnesium supplementation for the treatment of magnesium deficiency has been studied in detail. However, the proven and well-documented oral magnesium supplementation has become questioned in the recent years through intensive marketing for its transdermal application (e.g., magnesium-containing sprays, magnesium flakes, and magnesium salt baths). In both, specialist and lay press as well as on the internet, there are increasing numbers of articles claiming the effectiveness and superiority of transdermal magnesium over an oral application. It is claimed that the transdermal absorption of magnesium in comparison to oral application is more effective due to better absorption and fewer side effects as it bypasses the gastrointestinal tract.

## 1. Introduction

Magnesium has been recognized as a cofactor for more than 300 enzymatic reactions, where it is crucial for adenosine triphosphate (ATP) metabolism. Magnesium is required for DNA and RNA synthesis, reproduction, and protein synthesis. Moreover, magnesium is essential for the regulation of muscular contraction, blood pressure, insulin metabolism, cardiac excitability, vasomotor tone, nerve transmission and neuromuscular conduction. Imbalances in magnesium status—primarily hypomagnesaemia as it is seen more often than hypermagnesemia—might result in unwanted neuromuscular, cardiac or nervous disorders.

Severe hypermagnesemia or magnesium intoxication appears very seldom in human disease. Such conditions only occur in severe renal insufficiency or iatrogen. However, clinical symptoms are observed more frequently in magnesium-deficient and -insufficient patients in internal medicine. Magnesium deficiency is not uncommon among the general population: its intake has decreased over the years, especially in the Western world. Hypomagnesaemia is defined as serum magnesium concentration <0.75 mmol/L. Early signs of magnesium deficiency are non-specific and include loss of appetite, lethargy, nausea, vomiting, fatigue, and weakness. More pronounced magnesium deficiency presents with symptoms of increased neuromuscular excitability such as tremor, carpopedal spasm, muscle cramps, tetany and generalized seizures. Hypomagnesemia can cause cardiac arrhythmias including atrial and ventricular tachycardia, prolonged QT interval and torsades de pointes (see Table 1).

Hypomagnesaemia is frequently associated with other electrolyte abnormalities such as hypokalemia and hypocalcaemia. Conditions that may lead to hypomagnesemia include alcoholism, poorly-controlled diabetes, malabsorption (e.g., Crohn’s disease, ulcerative colitis, coeliac disease, short bowel syndrome, Whipple’s disease), endocrine causes (e.g., aldosteronism, hyperparathyroidism, hyperthyroidism), renal disease (e.g., chronic renal failure, dialysis, Gitelman’s syndrome) and medication use. A variety of drugs including antibiotics, chemotherapeutic agents, diuretics and proton pump inhibitors can cause magnesium loss and hypomagnesemia. In addition, magnesium deficiency exacerbates potassium-mediated arrhythmia, in particular in the presence of digoxin intoxication [1].

Magnesium compounds are widely used as medicinal and dietary supplements. The effectiveness of oral magnesium supplementation for the treatment of magnesium deficiency is beyond controversy [1,2,3,4,5]. Furthermore, a recent meta-analysis identified that a supplement of >370 mg magnesium/day shows greater efficacy than a lower dose in improving blood pressure and that magnesium supplementation gives a dose dependent response with regards to blood pressure [4].

In both, specialist and lay press as well as on the internet, there are increasing numbers of articles claiming the effectiveness and superiority of transdermal magnesium over an oral application. Transdermal magnesium application should be the ultimate way to replenish cellular magnesium levels since every cell in the body bathes in it. It passes directly into the tissues via the skin, where it should quickly be transported to cells throughout the body. Furthermore, the transdermal absorption of magnesium in comparison to oral application is presented as being more effective on the one hand due to nearly 100% absorption, and as presenting fewer side effects on the other hand as it is bypassing the gastrointestinal tract [6,7]. Scepticism based on ignorance impairs scientific evaluation as much as claims based on excessive faith.

## 2. Magnesium Absorption Transdermal

The skin is the largest organ of the body, covering about 1.8 m^2^ and comprising approximately 10% of the total body mass of an average person. The primary function of the skin is to provide a barrier between the body and the external environment. This barrier protects against the permeation of ultraviolet (UV) radiation, chemicals, allergens and microorganisms, in addition to the loss of moisture and body nutrients [8]. This means that the absorptive capacity of healthy skin for substances from the outside is very limited. This becomes evident particularly in the limited application for topical drugs. To get through the skin, a substance must penetrate the epidermis or has to be absorbed by sweat glands or hair follicles. The stratum corneum is the outermost layer of the epidermis consisting of dead cells (corneocytes). This layer is composed of about 15 to 20 layers of flattened cells with no nuclei and cell organelles. Their cytoplasm shows filamentous keratin. These corneocytes are embedded in a lipid matrix composed of ceramides, cholesterol, and fatty acids. The stratum corneum functions to form a water-repellent barrier to protect underlying tissue from infection, dehydration, chemicals and mechanical stress [9]. Overcoming this layer in significant quantities is only possible for lipophilic substances. In magnesium chloride solution, magnesium is present in ionized form and therefore not able to penetrate a lipophilic layer. In addition, the radius of the hydrated magnesium ion (86 pm) has been reported to be 400 times higher than its dehydrated form, leading to the assertion that it is almost impossible for magnesium ions to pass through biological membranes [2].

Therefore, cellular magnesium uptake is only being carried out by specific magnesium transporters and not by diffusion. However, since dead cells of the upper skin layer do not contain functional magnesium transporters, which have not yet been identified in detail, magnesium absorption may be possible only at the small area of sweat glands and hair follicles. A recently published study showed that magnesium ions can penetrate the stratum corneum in a concentration and time dependent manner which is significantly facilitated by hair follicles. However, hair follicles and sweat glands constitute only 0.1% to 1% of the skin surface. Even if a substance is absorbed in this area, the question of the clinical relevance of absorbed amounts needs to be addressed. In the study that examined the permeation of topically applied magnesium no information is given on the quantity of absorbed magnesium [10,11].

## 3. Effectiveness of Transdermal Magnesium Absorption

One of the first studies on transdermal magnesium absorption was published by the naturopathic doctor and founder of the American Holistic Medical Association Norman Shealy, M.D. Ph.D in 2000. He was an early advocate for the particular benefits of transdermal applications of magnesium [12]. Shealy argued that a magnesium deficiency can be compensated by transdermal application within 4 to 6 weeks, whereas an oral supplementation is effective only after 4 to 12 months. A full publication of this comparative study could not be found. Only an abstract for a conference was published not showing any additional data to substantiate this statement [7].

Another study that is often cited for proving that transdermal magnesium absorption offers a simple, cost effective and efficient methodology to increase cellular magnesium levels was a trial that took place over a 12-week period and involved a total of nine patients aged between 22 and 69 years (only weak statistical power). Following provision of a hair sample, although known to be only less representative for total body magnesium handling, for analysis of mineral content each patient tested was instructed to apply 20 sprays of magnesium oil. The original treatment consisted of the daily spray, anywhere on the body, as well as a 20-min foot soak using 100 mL magnesium oil (using a simple water footbath) twice weekly. At the end of 12-week treatment a further hair analysis was conducted. After transdermal applications for 12 weeks all patients except one had a significant increase in cellular magnesium ranging from −7.1% to 262%. One patient ceased application prematurely, three weeks before the final analysis. Overall an average increase of 59.7% in hair was observed. No data on serum magnesium concentration was available [6].

A study of similar questionable quality for proving transdermal magnesium absorption is an examination of Waring from the University of Birmingham, United Kingdom, where 19 subjects underwent a full body bath (temperatures 50–55 °C) for 7 days in a solution of magnesium sulphate (Epsom salt) for 12 min. Blood samples were taken before the first bath, at 2 h after the first bath and at 2 h after the seventh consecutive bath. Baths were taken daily at the same time for 7 days for the experiment. Urine samples were collected before the first bath and then 2 h after the first bath and at all subsequent baths. Urine samples were also taken 24 h after the last bath. All urine samples were corrected for creatinine content. Of the 19 subjects, all except three showed a rise in magnesium concentrations in plasma, though this was small in some cases. The values before the first bath had a mean of 104.68 ± 20.76 ppm/mL; after the first bath, the mean was 114.08 ± 25.83 ppm/mL. The continuation of bathing for 7 days in all except two individuals gave a rise to a mean of 140.98 ± 17.00 ppm/mL. Prolonged soaking in Epsom salts therefore increases blood magnesium concentrations. The measurement of magnesium levels in urine showed a rise from the control level, mean 94.81 ± 44.26 ppm/mL to 198.93 ± 97.52 ppm/mL after the first bath. Those individuals where the blood magnesium levels were not increased had correspondingly large increases in urinary magnesium showing that the magnesium ions had crossed the skin barrier and had been excreted via the kidney, presumably because the blood levels were already optimal. Generally, urinary magnesium levels 24 h after the first bath fell from the initial values found after day 1 (mean 118.43 ± 51.95) suggesting some retention of magnesium in tissues after bathing as blood levels were still high. The measurement of magnesium levels in urine 24 h after the seventh bath gave values almost back to control levels. However, this study has only been published on the internet—on the commercial site of the Epson-salt-council—but not in a scientific, peer-reviewed journal [13].

**Figure 1:**
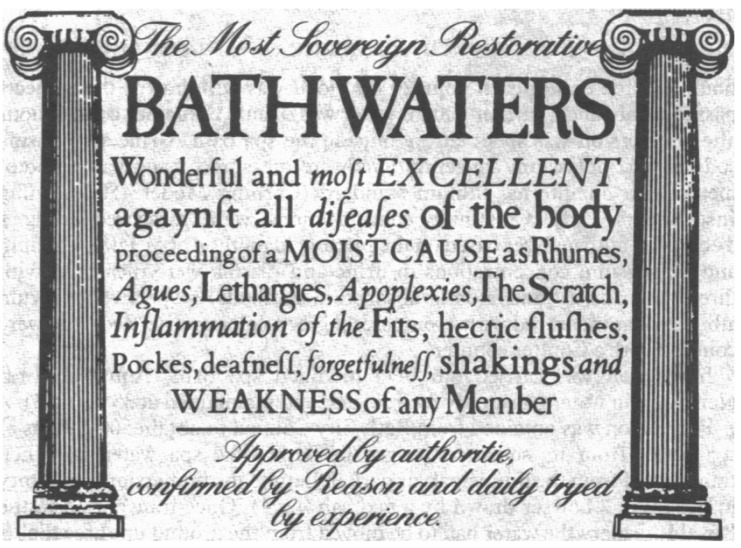
Eighteenth century bath card [16].

The most plentiful source of biologically available magnesium, however, is the hydrosphere (i.e., oceans and rivers). The Dead Sea, the deepest and most saline lake on earth, has been known from biblical times for its healing properties and the effectiveness of bathing in the Dead Sea is well known (Figure 1). In the sea, the concentration of magnesium is about 55 mmol/L and in the Dead Sea as an extreme example, the concentration of magnesium is reported to be 198 mmol/L and has steadily increased over time [14,15]. In comparison, the typical human serum magnesium concentration is only ~0.8 mmol/L [1]. Thus, there is a substantial gradient into the human body.

Near-drowning in the Dead Sea is expected to result in severe electrolyte abnormalities and respiratory failure. This suggests that magnesium does not pertain to the topic of dermal absorption. In one cohort study, the data were abstracted from the archives of Soroka University Medical Center. The cohort comprised 69 patients who nearly drowned in the Dead Sea. There were two major manifestations of near-drowning in the Dead Sea: electrolyte imbalance and acute lung injury. Serum calcium, magnesium and phosphorus (but not sodium, potassium and chloride) were abnormal in most patients. Median serum electrolyte levels (and range) on admission were 10.9 mg/dL (9–24) for calcium, 4.3 mg/dL (1–30) for magnesium, and 4.1 mg/dL (2–9) for phosphorus. These levels quickly normalized with forced diuresis within 24 h. Acute lung injury—namely, hypoxic bilateral pneumonitis—occurred in 29 patients. Mechanical ventilation was required in 11 patients. Sixty-five patients recovered fully, while the remaining four had minor sequelae [17].

The penetration of electrolytes through the human skin was measured in healthy volunteers and in psoriatic patients after bathing in the Dead-Sea (Dead Sea water balneotherapy) or in simulated bath-salt solutions. Significant increases in the levels of serum Bromine, Rubidium, calcium and zinc were noticed only in the psoriatic patients after daily bathing in the Dead-Sea for a four-week regimen. Guinea-pigs “bathed” in simulated Dead-Sea bath-salt solutions containing radionuclides of calcium, magnesium, potassium and Br. Traces of each radionuclide were detected in the blood and in some internal organs after 60 min of bathing. The radionuclides showed a physiological pattern in their organ distribution. Even though the whole investigation was carried out in hypertonic solutions, there is a definite penetration of salts through healthy (human and guinea-pig) and damaged (psoriatic) epidermis [17]. However, Dead Sea water balneotherapy does not lead to the worsening of blood pressure. Substantial ingestion of Dead Sea water (generally in unusual near-drowning cases) is toxic and can result in cardiac rhythm disturbances because of electrolyte concentration abnormalities. A laboratory analysis of Dead Sea mud did not reveal mineral concentrations that could represent a health concern for their intended use [18].

Eight normal subjects were immersed in Bath spa water for two hours and the renal, haematological, and cardiovascular responses were compared with those in the control periods before and after immersion. Immersion in Bath spa water resulted in highly significant diuresis compared with the control state before immersion (*p* < 0.01). The mean (SEM) total excess volume of water excreted as a result of immersion over the 2-h period was 510 (85) ml. A twofold increase in sodium excretion resulted from immersion (the rate before immersion was 86 (15) pmol/min and 170-8 (29) pmol/min during the second hour) (*p* < 0.01). A significant increase in potassium excretion was also observed on immersion and was of the same amount as the sodium excretion (from 78-6 (19) pmol/min before immersion to 156-2 (28) pmol/min by the second hour) (*p* < 0.01). A mean loss of weight of 0-53 (0–14) kg occurred. This corresponds with the losses of water from diuresis and also some loss from sweating. No significant change was seen in creatinine clearance throughout immersion, which ranged from mean values of 109 to 121 ml/min. As a sign that an uptake of magnesium by the healthy human skin while bathing is not possible or if so, only very limited no change occurred in the plasma concentrations of electrolytes, calcium, phosphate, or magnesium after 2 h bathing (35 °C). A significant fall in plasma albumin concentration was observed (from 44-5 to 41-5 g/L) (*p* < 0.001) [16].

Extensive studies of the Israel army with a magnesium-containing skin protectant lotion (IB1) showed that magnesium is not absorbed through the skin, as tested in preclinical animal studies in pigs in contrast to the lipophilic nature of sulfur mustard and VX, potent chemical warfare agents that penetrate rapidly through the skin, causing severe prolonged injuries and sometimes death [19].

The topical skin protectant lotion (IB1) containing magnesium was tested in a human study. Preclinical studies in several animal models have proven the protective efficacy of IB1. In a randomized, placebo-controlled phase I clinical study it was examined whether a magnesium-rich lotion, after repeated topical application, leads to changes in serum magnesium concentrations in 34 healthy volunteers. The 34 subjects administered 10 mL of magnesium-rich lotion or placebo lotion three times daily over a period of three days. The study tested the safety of repeated applications, including ruling out the transdermal permeation of magnesium, which may lead to a dangerous blood magnesium level, since the lotion contains magnesium sulphate. Other objectives included the detection of dermatological adverse effects, the assessment of application convenience, and the effect on daily activities. Importantly, no serious adverse effects were recorded and the lotion did not interfere with daily tasks. There were no significant differences in magnesium levels between the placebo and the study groups in any of the applications. No toxic levels of magnesium were found in either group [20].

Also in a study on isolated human cadaver skin, no significant difference could be found for the skin penetration of magnesium from solutions containing magnesium chloride (MgCl_2_). The purpose of this study was to compare the passive permeation of magnesium across human skin from pharmaceutical grade MgCl_2_ formulated in cream to that of pharmaceutical grade MgCl_2_ in solution. The transdermal permeation efficiency of magnesium from MgCl_2_ cream I and MgCl_2_ cream II was studied across skin compared to the positive control MgCl_2_ solution and the negative control phosphate buffer solution. The cream or MgCl_2_ solution equivalent to 2.76 mg of magnesium was applied per 2.52 cm^2^ of skin and mounted on diffusion cell. Samples were collected after 1, 2, 3, 4, 5 and 24 h and analyzed using atomic absorption spectroscopy at 285 nm. The experiments were performed in triplicates. The results were analyzed using unpaired *t*-test. The cumulative magnesium permeation from magnesium cream I, magnesium cream II, MgCl_2_ solution, and phosphate buffer across human skin after 24 h were found to be 29.79 ± 13.92, 24.53 ± 9.98, 6.18 ± 1.36, and 5.62 ± 1.83 μg respectively. This study showed that a magnesium cream leads to statistically significant (*p* < 0.05) magnesium permeation compared with the two control solutions; Magnesium cream I showed greater magnesium permeation than magnesium cream II, but the difference was not statistically significant. The MgCl_2_ solution showed a similar result to that of phosphate buffer. A formulated magnesium cream was able to successfully deliver the magnesium of pharmaceutical grade MgCl_2_ across human skin. Transdermal magnesium may play an important role treating symptoms of sub-optimal magnesium status. However, further in vitro and animal studies are warranted to establish the efficacy of formulations [21].

A recent pilot study set out to determine whether magnesium in a cream could be absorbed transdermally in humans to improve magnesium status (6). Current formulations include magnesium oils and transdermal creams, from which the magnesium may be absorbed across the skin and into the systemic circulation. This cream was manufactured, in the course of research and development, for the Center for Magnesium Education & Research, by Urist Cosmetics of Vancouver, B.C. Canada. For full ingredients, see S1 text ingredients list of company.

However, in contrast to the gastrointestinal epithelium, a primary function of the skin is to act as a barrier, which normally restricts the absorption of exogenous chemicals into the body. In this single blind, parallel designed pilot study participants (*n* = 25 four participants dropped out, aged 34.3 ± 14.8 years, height 171.5 ± 11 cm, weight 75.9 ± 14 Kg) were randomly assigned to either a 56 mg/day magnesium cream (*n* = 11) or a placebo cream group (*n* = 10) for two weeks. Magnesium serum and 24 h urinary excretion were measured at baseline and at 14 days of the intervention. Food diaries were recorded for eight days during this period. Mg test and placebo groups' serum and urinary magnesium did not differ at baseline. After the Mg^2+^ cream intervention there was a clinically relevant increase in serum magnesium (0.82 to 0.89 mmol/L, *p* = 0.29) that was not seen in the placebo group (*n* = 10) (0.77 to 0.79 mmol/L), but this difference was only statistically significant (*p* = 0.02)) in a subgroup of non-athletes (*n* = 20). Magnesium urinary excretion increased from baseline slightly in the Mg^2+^ group but with no statistical significance (*p* = 0.48). The Mg^2+^ group showed an 8.54% increase in serum Mg^2+^ and a 9.1% increase in urinary Mg^2+^ while these figures for the placebo group were smaller, i.e., +2.6% for serum Mg^2+^ and −32% for urinary Mg^2+^. In the placebo group, both serum and urine concentrations showed no statistically significant change after the application of the placebo cream. In this two-week pilot study, transdermal delivery of 56 mg Mg/day (a low dose compared with commercial transdermal Mg^2+^ products available) showed a larger percentage change in both serum and urinary markers from pre to post intervention compared with subjects using the placebo cream. However, based on this data from the original abstract, Kass et al. only show a slight increase of serum magnesium via transdermal magnesium-containing cream [22].

## 4. Conclusions

Numerous studies demonstrate the effectiveness of oral therapeutic or preventive magnesium supplementation. Thus, an adequate magnesium supply is important for healthy pregnancy and lactation, as well as in patients with diabetes and prediabetes. Magnesium supplementation is also useful when taking drugs such as diuretics and proton pump inhibitors. Based on the current studies it is extremely alarming if a successful treatment of magnesium fails by propagation of transdermal magnesium, a scientifically not yet proven form of magnesium application. We suggest that future research should focus on a larger number of human subjects given higher concentrations of, for example, a magnesium cream application administered for longer durations to investigate whether transdermal application may show a significant contribution to improvement in magnesium status. Magnesium might be able to get into the lymphatic system beneath the dermis and enter the circulatory system, bypassing the regulation through the GI tract and hereby increasing serum magnesium [23,24,25]. However, we cannot yet recommend the application of transdermal magnesium.

## Figures and Tables

**Table 1:** Magnesium: Deficiency signs and symptoms [1].

**General**	Anxiety, lethargy, weakness, agitation, depression, dysmenorrhea, hyperactivity, headache, irritability, dysacusis, low stress tolerance, loss of appetite, nausea, sleep disorders, impaired athletic performance.
**Musculature**	Muscle spasm, cramps in the soles of the feet, leg cramps, facial muscles, masticatory muscles, and calves, carpopedal spasm, back aches, neck pain, urinary spasms, magnesium deficiency tetany.
**Nerves/CNS**	Nervousness, increased sensitivity of NMDA receptors to excitatory neurotransmitters, migraine, depression, nystagmus, paraesthesia, poor memory, seizures, tremor, vertigo.
**Gastrointestinal tract**	Constipation.
**Cardiovascular system**	Risk of arrhythmias, supraventricular or ventricular arrhythmias, hypertension, coronary spasm, decreased myocardial pump function, digitalis sensitivity, torsade de pointes, death from heart disease.
**Electrolytes**	Hypokalaemia, hypocalcaemia, retention of sodium.
**Metabolism**	Dyslipoproteinemia (increased blood triglycerides and cholesterol), decreased glucose tolerance, insulin resistance, increased risk of metabolic syndrome, disturbances of bone and vitamin D metabolism, resistance to PTH, low circulating levels of PTH, resistance to vitamin D, low circulating levels of 25(OH)D, recurrence of calcium oxalate calculi.
**Miscellaneous**	Asthma, chronic fatigue syndrome, osteoporosis, hypertension, altered glucose homeostasis.
**Pregnancy**	Pregnancy complications (e.g., miscarriage, premature labor, eclampsia).
